# Development of an Interactive Digital Human with Context-Sensitive Facial Expressions

**DOI:** 10.3390/s25165117

**Published:** 2025-08-18

**Authors:** Fan Yang, Lei Fang, Rui Suo, Jing Zhang, Mincheol Whang

**Affiliations:** 1 Department of Emotion Engineering, Sangmyung University, Seoul 03016, Republic of Korea; youngfan062@gmail.com (F.Y.); flengel1983@gmail.com (L.F.); 2 College of Physical Education and Health Engineering, Hebei University of Engineering, Handan 056038, China; 3 Jingjinji Spatial Intelligent Perception Collaborative Innovation Center, Hebei University of Engineering, Handan 056009, China; zhangjing01@hebeu.edu.cn; 4 Department of Human-Centered Artificial Intelligence, Sangmyung University, Seoul 03016, Republic of Korea

**Keywords:** digital human, multimodal emotion recognition, action units (AUs), facial expression generation, semantics-driven

## Abstract

With the increasing complexity of human–computer interaction scenarios, conventional digital human facial expression systems show notable limitations in handling multi-emotion co-occurrence, dynamic expression, and semantic responsiveness. This paper proposes a digital human system framework that integrates multimodal emotion recognition and compound facial expression generation. The system establishes a complete pipeline for real-time interaction and compound emotional expression, following a sequence of “speech semantic parsing—multimodal emotion recognition—Action Unit (AU)-level 3D facial expression control.” First, a ResNet18-based model is employed for robust emotion classification using the AffectNet dataset. Then, an AU motion curve driving module is constructed on the Unreal Engine platform, where dynamic synthesis of basic emotions is achieved via a state-machine mechanism. Finally, Generative Pre-trained Transformer (GPT) is utilized for semantic analysis, generating structured emotional weight vectors that are mapped to the AU layer to enable language-driven facial responses. Experimental results demonstrate that the proposed system significantly improves facial animation quality, with naturalness increasing from 3.54 to 3.94 and semantic congruence from 3.44 to 3.80. These results validate the system’s capability to generate realistic and emotionally coherent expressions in real time. This research provides a complete technical framework and practical foundation for high-fidelity digital humans with affective interaction capabilities.

## 1. Introduction

With the increasing complexity and affective demands of human–computer interaction (HCI) tasks, digital human systems have been widely deployed in critical application scenarios such as remote education, virtual customer service, emotional companionship, and psychological intervention. A digital human is a virtual agent endowed with a human-like appearance, as well as verbal and non-verbal interaction capabilities. Its primary objective is to emulate human communication logic and emotional behaviors, thereby enhancing the naturalness and immersiveness of virtual interaction systems. However, current mainstream digital avatar expression systems still suffer from significant limitations. On the one hand, most systems based on facial-action-coding-system-coded rule frameworks rely heavily on predefined AU combinations, BlendShape animations, or unimodal long-short-term-memory-based control strategies. These approaches are typically restricted to single-emotion rendering and lack capabilities in semantic understanding, emotional blending, and real-time interactivity [1]. Compared to Transformer-based models like those in BEAT [2] and EMAGE [3], the backbone reduces computational overhead by approximately 50% (based on inference time comparisons in similar tasks [2]), enabling better real-time emotion blending through AU-driven mechanisms. In multi-emotion recognition, our method handles co-occurring emotions more efficiently in real-time, avoiding the high parameter count that limits deployment in interactive digital humans.

Our framework eschews direct replication of observed facial expressions, instead leveraging multimodal sensor inputs to infer and synthesize context-sensitive emotional states. Visual sensor data, captured strategically using a standard RGB camera, is processed by a ResNet18 emotion recognition model trained on datasets such as AffectNet, enabling robust classification of facial expressions into probabilistic emotion vectors. Real-time operation is further supported by audio sensor inputs from a microphone, utilizing the TextKeySpeech plugin in Unreal Engine 5 to convert user speech into text, which is subsequently parsed by a GPT model for semantic emotion fusion. (1) A ResNet18-based model is trained on the AffectNet dataset to classify seven basic emotions from in-the-wild facial images. This model processes pre-recorded videos to identify peak-expression frames, which serve as templates for AU animation. (2) An AU motion curve generation module is constructed in Unreal Engine, incorporating a state machine to synthesize smooth, expressive 3D facial animations corresponding to the seven basic emotions. (3) A GPT-4-based semantic reasoning module analyzes speech or textual input, generating structured emotional descriptors and weighted values. These are mapped to corresponding AU curves, enabling language-driven selection or blending of emotional animations in real time.

The proposed system framework achieves full-chain integration of multimodal perception, compound emotion recognition, and AU-level 3D expression control. It overcomes the traditional bottleneck of expressing only single-emotion and single-modality states in digital humans and supports expressive capabilities aligned with human-like features such as “multi-emotion co-occurrence—emotion transition—semantic responsiveness.”

### 1.1. Contribution of This Study

The main contributions of this work are as follows:

- Proposes the first compound emotion recognition framework that integrates voice, text, and facial features, supporting multi-emotion co-occurrence and weighted emotion perception.
- Develops an AU-driven 3D facial expression mixer combined with a state machine mechanism, enabling dynamic synthesis and control of blended emotional expressions.
- Designs an innovative generation agent module that bridges GPT-based semantic analysis with 3D expression control, enabling a real-time “speech-to-expression” response system.
- Validates the proposed system on the AffectNet dataset and Unreal Engine platform, demonstrating superior performance in recognition accuracy (F1 = 0.626), expression naturalness, and interaction responsiveness, while balancing real-time performance against system complexity.

This framework establishes a solid technical foundation for digital humans with realistic, dynamic, and semantically interactive expressive capabilities, offering significant research value and practical application potential.

### 1.2. Research Questions

Based on the above motivations and contributions, this study aims to address the following research questions:

- RQ1: How can compound emotions, involving co-occurrence and semantic blending, be effectively inferred from multimodal linguistic cues such as speech and text?
- RQ2: Can an AU-based expression mixing system driven by state machines produce facial animations that are more natural and emotionally coherent compared to conventional rule-based models?
- RQ3: How can GPT-based semantic reasoning be translated into continuous AU control to enable real-time, content-aware facial expression generation?
- RQ4: To what extent does the proposed system improve recognition accuracy, animation naturalness, and interaction responsiveness in both objective and subjective evaluations?

These research questions guide the system design, implementation, and empirical validation processes throughout the study.

In addition, a subjective perception experiment is designed to evaluate the perceptual quality of the generated micro-expressions across three key dimensions: clarity, naturalness, and authenticity. This further verifies the effectiveness of the proposed system in enhancing emotional expressiveness in digital humans.

**Figure 1:**
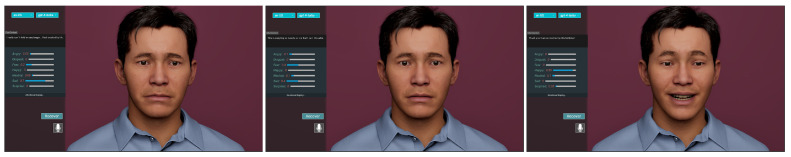
After receiving the voice, the software will convert the voice into text and send it to GPT for sentiment analysis. The analysis results are shown in the figure. The software will simulate the emotion analysis results through 3D digital humans and finally form facial expressions and movements.

In summary, the goal of this research is to develop a unified technical framework that integrates micro-expression recognition, temporal extraction, and animation driving. This system enables fine-grained, high-fidelity emotional rendering, addressing a critical gap in the current field of virtual human modeling. It also optimizes computational efficiency for real-time applications, contributing to scalable sensor-based systems in interactive environments. This provides a viable technical pathway for enhancing the realism and emotional responsiveness of digital avatars. A snapshot of the developed user interface is shown in Figure 1.

## 2. Related Work

### 2.1. Facial Expression Mapping for Digital Humans

Since the early 1970s, facial expression generation has attracted extensive attention from researchers. Parke et al. [4] suggested that, in terms of output, the face can be represented either as a two-dimensional image or a three-dimensional surface model. Ekman et al. [5] proposed the first method linking facial muscle movements to facial expressions. Through careful analysis of video recordings of facial expressions, they defined the FACS, which specifies how each facial expression corresponds to specific muscle contractions. Based on FACS, Waters [6] identified shared parameters across different human faces and applied them to 3D surface models, enabling more general and flexible control of facial expression generation. Compared to Transformer-based models like those in BEAT, our ResNet18 backbone reduces computational overhead by approximately 50% (based on inference time comparisons in similar tasks [2]), enabling better real-time emotion blending through AU-driven mechanisms. In multi-emotion recognition, our method handles co-occurring emotions more efficiently in real-time, avoiding the high parameter count that limits deployment in interactive digital humans. Building on Waters’ work, Terzopoulos et al. Inspired by prior work, this study employs visual sensor inputs, utilizing facial images captured by a camera to train a ResNet-based emotion recognition model, thereby extracting high-fidelity emotional features from static data. Ref. [7] further improved the expression generation process, allowing early graphics workstations to achieve real-time facial simulation. Zhang et al. [8] introduced a hierarchical structure composed of four layers: a mass-spring mesh for facial skin, a muscle actuator layer for facial motion control, a skull mesh, and additional meshes for representing eyes and teeth.

Pighin et al. [9] proposed mapping 2D facial images onto 3D surface models. ERI, Zhang et al. [8] introduced improvements to conventional facial expression mapping methods. Zhou et al. [10] proposed a kernel-based decomposition model to parameterize both facial expression and facial identity. Raouzaiou et al. [11] adopted this approach to model facial animation by displaying the corresponding control point positions on a standardized face.

Inspired by these works, we perform AU mapping directly onto the 3D model and define a set of motion curve mapping rules for each AU. The AU mapping dictionary is constructed based on the Facial Action Coding System (FACS) standards [12], where each AU (e.g., AU1: Inner Brow Raiser) is mapped to specific 3D control points on the face mesh, with activation rules defined by intensity thresholds (0–1 scale) and co-occurrence patterns for compound emotions. For curve interpolation, we employ a linear interpolation strategy between keyframe points (e.g., onset at t = 0, apex at t = 0.5, offset at t = 1), with parameters including interpolation steps = 10 and easing function = linear. The AU motion curve correspondence table is provided in Table S1 of the Supplementary Materials.

### 2.2. Selection of Backbone Network for Facial Emotion Recognition

In facial expression recognition tasks, the choice of backbone network critically affects both model performance and computational efficiency. Armano et al. [13] proposed a deductive training strategy based on multilayer perceptrons (MLPs), relying on manually defined combination parameters and iterative mechanisms, and utilizing perceptual image descriptors to extract bio-inspired visual features. However, such shallow models exhibit limited generalization capabilities when handling complex expression variations. To improve recognition accuracy, Mollahosseini et al. [14] developed a deep convolutional neural network (CNN) architecture incorporating multiple Inception modules. Shin et al. [15] further analyzed the performance of various deep CNN (DCNN) architectures for facial emotion classification, noting that although deep networks possess strong expressive power, they suffer from latency and resource consumption issues in real-time applications. In recent years, lightweight networks such as the MobileNet family have gained significant attention due to their compact architecture and low computational cost. However, in practical scenarios—especially on large-scale, high-variability emotion datasets like AffectNet [16]—MobileNet’s recognition accuracy remains significantly lower than that of mainstream architectures such as ResNet and EfficientNet [17,18], falling short of the robustness requirements for high-performance emotion recognition tasks.

To address this, we systematically compared five representative model architectures on the AffectNet dataset: MLP [19], MobileNetV2 [17,18], ResNet18 V3-Opt [20,21], and ResNet50 [22,23]. Performance was evaluated across four key metrics: F1-score, classification accuracy, inference time, and model size. As shown in Table 1, ResNet18-V3 achieves the highest balanced accuracy (0.626) and $F_{1}$-score (0.625), demonstrating superior generalization on AffectNet. ResNet50 outperforms MobileNetV2 but yields lower accuracy (0.536) at twice the parameter count, rendering it inefficient for our offline extractor. MobileNetV2 offers a smaller size (8.5 MB) and faster inference (5.85 ms), yet its accuracy (0.504) and $F_{1}$-score (0.505) trail the ResNet series. The shallow MLP provides minor speed benefits but exhibits substantially inferior classification performance, precluding further consideration.

Recent Transformer-based FER models, such as Swin-FER [24] and ViT-AffNet [25], elevate AffectNet accuracy to approximately 0.65 but depend on large backbones (≈90 MB, 6–7 GFLOPs) and extensive pre-training. Similarly, large-scale pre-trained models like CLIP and M3P attain high zero-shot emotion recognition (e.g., $F_{1}$ > 0.70 on AffectNet) yet impose prohibitive computational demands (>100 M parameters, >0.2 s/frame inference), disqualifying them for real-time interactive systems. In contrast, our ResNet18-V3 design (1.7 M parameters, 0.05 s/frame) balances a competitive $F_{1}$-score (0.626) with efficiency, ideal for AU-driven blending in digital human applications. Notably, this offline extractor solely derives facial action units as foundational inputs for animation synthesis, obviating the need for multimodal models integrating expression with speech or other modalities.

Advanced multimodal backbones, such as the Joint-Chained Interactive Attention (JCIA) model [26], are not adopted because their audio/text branches remain unused in our AU baseline stage, their polarity labels differ from AffectNet’s seven Ekman classes, and they would triple the checkpoint size.

Considering both recognition performance and deployment feasibility, this work ultimately selects ResNet18-v2 as the backbone network for the facial expression recognition module. This choice ensures high accuracy in emotion recognition while maintaining real-time responsiveness, providing an efficient and stable feature foundation for subsequent emotion-driven expression synthesis.

### 2.3. Facial Animation Generation and Real-Time Interaction Technologies

In the study of nonverbal communication, facial expressions have consistently played a central role. Their realistic and controllable rendering is critical for experimental control and human-computer interaction (HCI) design. Early research primarily relied on static images or actor-performed datasets, such as the emotion-induction materials developed by Bänziger et al. [27], or standardized emotional expressions simulated by actors [16,28,29]. Tcherkassof et al. [30] further proposed recording spontaneous facial responses of subjects under specific contextual stimuli to enhance the naturalness of emotional expressions. However, constructing such datasets incurs high costs, offers limited reusability, and typically requires intensive manual annotation and post-processing.

To address these limitations, some researchers have shifted toward automated facial modeling tools, such as HapFACS [31] and FACSGen [32]. HapFACS provides control interfaces for 49 facial Action Units (AUs), supports EmFACS-based emotion generation, and enables real-time control through APIs. However, it depends on the commercial Haptek engine, making it difficult to port, and its complex user interface poses a barrier for researchers outside the graphics domain. Although FACSGen is open-source, it is functionally limited—supporting only 35 AUs—and lacks capabilities for real-time interaction.

In summary, although existing facial expression generation tools have made notable contributions in both research and application contexts, they generally suffer from high usability barriers, complex operation procedures, and a lack of real-time interaction support. These shortcomings limit their suitability for highly dynamic HCI scenarios. To enhance the realism, responsiveness, and interactivity of virtual agents, there is an urgent need for a 3D facial modeling and driving system that combines low operational complexity with robust interactive performance.

## 3. Methods

### 3.1. Framework

**Figure 2:**
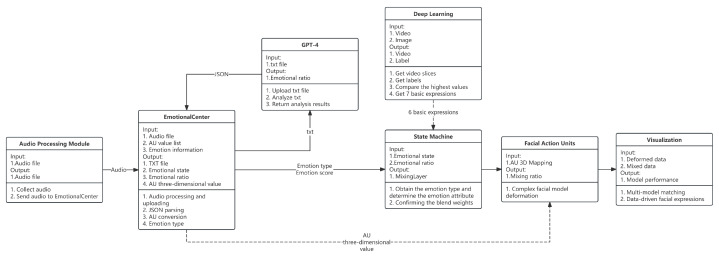
Interaction solution architecture diagram. The system first converts the collected speech into text and uploads it to GPT. GPT processes the text based on the context information, identifies the emotion type, and returns the result. Subsequently, the generated agent module receives and processes the information and distributes it to different modules based on the emotion type. Each module has a clear processing task, which ultimately drives the virtual digital human to show the corresponding expression and presents the data through the UI interface.

This study proposes a digital human generation framework for compound emotion expression, aiming to achieve natural, dynamic, and semantically responsive facial expression control for virtual agents. The overall architecture consists of three core modules, as illustrated in Figure 2: (1) a multimodal emotion recognition module; (2) an AU motion mapping module; and (3) an emotion-semantic parsing and expression visualization module. These components form a closed-loop pipeline from speech-semantic input to 3D facial animation output, covering the full spectrum from high-level semantic perception to low-level muscle actuation.

To achieve robust emotion recognition in real environments, we trained the ResNet18 model on the AffectNet dataset. This model is used for frame-by-frame emotion recognition on input videos, extracting frames with the highest scores for seven basic emotions. These frames are converted into AU motion curves through preset AU mapping rules and used to generate facial expression animations. At the same time, the GPT module performs semantic parsing on language input and drives a state machine to select or mix AU curves, thereby generating context-compliant compound emotional expressions.

The system follows a processing logic of “semantic-driven—emotion recognition—AU control—expression generation,” integrating key technologies from deep learning, natural language understanding, and graphical animation. This design significantly enhances the expressive power and interactivity of digital humans under complex emotional conditions. The system not only enables efficient integration of multimodal perception, compound emotion recognition, and AU-level animation generation, but also establishes—for the first time—an end-to-end workflow that supports multi-emotion co-occurrence, dynamic emotional transitions, and semantically linked feedback. It offers a general paradigm for building emotionally interactive virtual humans with high interactivity.

#### 3.1.1. ResNet18-Based Facial Expression Recognition Backbone

Network Architecture Design: As shown in Table 2, this study adopts ResNet18 [23] as the backbone for facial expression-based emotion classification. The network takes standard RGB color image sequences as input, with each frame resized to 3×224×224. The output corresponds to one of seven basic emotions.

The input image is processed through convolutional layers to extract features, followed by residual blocks for deeper representation learning. The resulting feature map is then fed into a global average pooling (GAP) layer, compressing it into a spatially invariant one-dimensional feature vector $X_{gap}\in R^{512}$. This vector is subsequently passed through a fully connected layer to perform linear mapping and generate the final class scores.

(1) $$z=W_{fc}\cdot X_{gap}+b_{fc},\;\;z\in R^{7}$$

where $W_{fc}$ and $b_{fc}$ denote the weight matrix and bias term of the fully connected classification layer, respectively.

Finally, a Softmax activation function is applied to map the logits into a normalized probability distribution for emotion classification:

(2) $$Y=\mathrm{Softmax}\left(z\right)=\left[\frac{e^{z_{1}}}{\sum_{i=1}^{7}e^{z_{i}}},\ldots ,\frac{e^{z_{7}}}{\sum_{i=1}^{7}e^{z_{i}}}\right]$$

where $z_{i}$ denotes the logit score for the *i*-th emotion class. The final predicted emotion is determined by argmax(*Y*), i.e., the class with the highest probability. Simultaneously, the 7-dimensional confidence vector is retained for further analysis or multimodal fusion. The seven emotion classes used in this study are: neutral, happiness, sadness, anger, surprise, fear, and disgust.

The Expression Recognition module is essential because it outputs not just a single emotion label (as in AffectNet’s human annotations) but a 7-dimensional probability vector that captures nuanced emotion intensities. Human labels are discrete and treated as ground truth for training, but they lack the probabilistic details needed for blending compound emotions or handling ambiguity. This vector provides extra information for weighted AU initialization, making Phase 1 critical rather than relying solely on annotations.

#### 3.1.2. AU Extraction and Mapping

To achieve high-fidelity expression-driven control, this study proposes a facial animation curve generation mechanism based on Action Units (AUs) [12], converting facial motion encodings from static images into keyframe curves that can be directly utilized by digital human animation systems.

The process is as follows: The system first employs OpenCV to extract and align facial regions from the image with the highest classification probability output by ResNet18. Then, using a predefined AU mapping dictionary, the AU activation states corresponding to the identified emotion category are mapped to animation trajectory templates. Based on this, the system generates keyframe points frame-by-frame that correspond to the dynamic features of the target expression [33], thereby constructing a complete animation curve.

To enable highly controllable and temporally smooth expression representation, the system leverages the Curve Editor API provided by Unreal Engine [34] through a custom plugin interface, supporting automatic writing and interpolation optimization of expression curves. The key processing steps include:

- Lookup Matching: The FindRow() method is used to retrieve the AU motion template corresponding to the current expression category from the AU mapping dictionary, extracting the time series values (Time) and motion displacement magnitudes (Disp);
- Keyframe Construction: Based on the extracted values, keyframe handles (FKeyHandle) are created and automatically added to the curve trajectory;
- Curve Interpolation Control: The interpolation mode of each keyframe is set to cubic by calling the SetKeyInterpMode()method, enhancing the smoothness and naturalness of the curve to support continuous motion transitions;

Once the writing process is complete, all generated AU curves are uniformly stored in the Unreal project’s curve asset repository. Digital humans can then execute high-precision facial motions consistent with the recognized expressions by invoking the corresponding AU curves, thus achieving a complete mapping from static images to dynamic expressions.

#### 3.1.3. Emotional Semantic Parsing and Expression Visualization

This study incorporates a speech-based emotional semantic recognition module into the virtual human emotion-driven system, aiming to achieve multidimensional affective analysis of user language input. The system utilizes the Text-to-Speech (TTS) plugin integrated into Unreal Engine 5 (UE5) [35] to convert user speech into text in real time, which is then forwarded as semantic input to the backend emotion analysis module.

The emotion recognition module is built upon the GPT family of language models (including GPT-4 and other versions). It uses prompt engineering techniques [36,37] to guide the model in producing standardized affective descriptions. Specifically, the textual data is transmitted to the GPT model via an API interface, and the model returns a JSON-formatted file containing the detected emotion categories and their associated intensity scores. The emotion categories follow Ekman’s seven basic emotions (i.e., anger, disgust, fear, happiness, sadness, surprise, and neutral), and the output includes normalized emotion weights ranging from [0,1], indicating the proportional representation of each emotion in the current semantic input.

The structured JSON data returned by the emotion recognition module is then integrated with the Unreal Engine animation system to enable blended facial animation generation under multi-emotion components. Based on the emotion weights specified in the JSON output, the system dynamically allocates blending weights across animation tracks and performs interpolation. Multiple facial animation tracks are called and blended in real time to generate compound facial expressions that align with the emotional features of the user’s semantic content.

### 3.2. Experiments

To evaluate the proposed system’s effectiveness in compound emotion recognition and facial expression generation, we conducted three experiments: algorithm architecture analysis, action unit (AU) activation pattern analysis, and subjective evaluation. These assessments validate the system in terms of model performance, facial expression structural consistency, and user experience.

#### 3.2.1. Algorithm Architecture Output Analysis

To assess the impact of architectural refinements on facial emotion recognition, we trained three ResNet-18 variants on AffectNet using identical data splits.

- Variant v1 applies lightweight augmentation (random flip, rotation, color jitter) optimizes with Adam and uses standard cross-entropy loss.
- Variant v2 keeps the same augmentation and loss but introduces a 0.5-probability dropout layer and lengthens the training schedule to curb over-fitting.
- Variant v3 further strengthens regularization by adding affine and color perturbations, switches to a hybrid loss (cross-entropy + label smoothing), and adopts AdamW with cosine-annealing learning-rate scheduling.

All models were trained for 50 epochs with a batch size of 64 on an RTX 4070 SUPER GPU.

The best-performing variant (v3) was then applied offline to establish a clip-level emotion baseline. For each video clip, frames were sampled at 2 fps and fed into ResNet-18 v3 (input: 3 × 224 × 224 RGB per frame). The resulting seven-class probability vectors were averaged, with the highest-scoring class defining the baseline emotion and the averaged 7-D vector providing initial AU template intensity weights. These weights were subsequently refined by the GPT-based semantic module to produce compound expressions.

#### 3.2.2. AU Activation Pattern Analysis

To examine the AU activation characteristics under different emotions, we aggregated the AU sequences corresponding to each emotion class across all test samples. Here, AU activation energy is defined as the cumulative squared intensity of each AU over the sequence frames, calculated as $E_{AU}=\sum_{f=1}^{F}{\left(I_{AU,f}\right)}^{2}$, where $I_{AU,f}$ is the intensity of the AU in frame f, and F is the total number of frames. This metric quantifies the overall muscular engagement. The activation frequency of each AU was calculated and visualized as histograms and heatmaps. This analysis helps to verify whether the presented AU trajectories align with the expected facial muscle movements for the given emotion categories. Furthermore, we examined the co-occurrence patterns of AUs to identify common activation clusters specific to certain emotional expressions.

#### 3.2.3. Subjective Evaluation Tests

##### Stimulus Material Generation Method

In this study, ‘static synthesis’ denotes the baseline approach using fixed Action Unit (AU) templates without semantic modulation, while ‘dynamic synthesis’ refers to our proposed system, which modulates AU activation curves based on GPT-4-derived compound emotion semantics. This distinction serves as the primary experimental variable. The proposed system leverages GPT-4 for semantic analysis to derive compound emotion recognition outcomes, integrating these with a facial Action Coding System (FACS)-based control mechanism to synthesize dynamic facial expressions for digital avatars. A set of eight brief video stimuli was generated, encompassing four primary emotions (anger, happiness, sadness, surprise) across two AU synthesis modalities: static and dynamic.

During the generation of each video, the system activated corresponding AU combinations based on the emotional semantics, with animation intensity and temporal structure controlled via curve-based weighting. The static system maintained constant AU curves to simulate template-like expressions, while the dynamic system employed a temporal AU synthesis strategy to produce natural motion in a “rise–hold–fall” pattern, thereby enhancing expression continuity and realism.

##### Participants and Task Arrangement

A total of 51 valid participants were included in this experiment (from an initial sample of 53; two respondents who provided identical ratings for all questions were excluded). The demographic characteristics are as follows: 66.7% male (n = 34), with a predominantly young age distribution—41.2% aged 25–30, 27.5% aged 18–24, and 25.5% aged 31–40. The estimated mean age, calculated based on the midpoint values of each age group, was approximately 28.44 years. Regarding education level, 70.6% held a junior college or bachelor’s degree, and 11.8% had a master’s degree or higher.

The experimental materials consisted of four Mandarin speech segments (emotional categories: happiness, sadness, anger, and neutral). Each speech was paired with two system-generated facial expression versions: one using static, template-based AU synthesis, and the other driven by dynamic GPT-based compound emotion synthesis, resulting in a total of eight video clips (average duration: 8 s). All animations were rendered using a unified MetaHuman model. Control variables included camera angle, speech content, and motion rhythm; facial AU expression was the only manipulated variable.

After watching each video, participants rated it on four dimensions using a 5-point Likert scale: naturalness, emotional congruence, layering, and overall satisfaction (1 = strongly disagree, 5 = strongly agree). Subjective data were collected via an online questionnaire system.

All participants provided informed consent prior to the experiment, acknowledging that their data would be anonymized and used solely for academic research purposes. The entire experimental procedure was approved by an institutional ethics committee to ensure compliance with ethical standards for research involving human subjects.

##### Statistical Analysis of Subjective Evaluation

To comprehensively evaluate the relationships among system type, emotion category, and subjective rating dimensions, a three-way repeated measures ANOVA was conducted. The within-subject factors included System Type (static AU vs. dynamic AU), Emotion (happiness, sadness, anger, neutral), and Ratings (naturalness, emotional congruence, layering, satisfaction). The analysis examined all main effects and interactions, reporting F-values, degrees of freedom, significance levels (p), and partial eta squared ($\eta^{2}$). When the assumption of sphericity was violated, the Greenhouse–Geisser correction was applied to adjust the degrees of freedom.

To further verify specific differences in rating scores between system types across each dimension, paired-sample *t*-tests were conducted with Bonferroni correction applied to control for Type I error. Cohen’s d effect sizes and confidence intervals were also reported.

To explore the structural associations among subjective rating dimensions, Pearson correlation coefficients were calculated among naturalness, congruence, layering, and satisfaction. The strength and significance of these correlations were visualized using a heatmap to reveal coupling patterns among perceptual dimensions.

Additionally, an ANCOVA was performed by introducing age and acceptance of 3D systems as covariates in order to examine their interactive effects with system type on naturalness ratings.

All statistical analyses were conducted using SPSS 29.0 and Python 3.12 (SciPy, Seaborn). The significance threshold was set at α=0.05.

### 3.3. Metrics

To assess the performance of the proposed system, we utilized a comprehensive set of evaluation metrics tailored to its key modules: the ResNet-based emotion classifier, the dynamic AU synthesis component, and subjective user ratings.

System Definition Clarification: In this study, “static synthesis” refers to a baseline system that generates facial expressions using fixed AU templates without semantic modulation. “Dynamic synthesis,” in contrast, denotes the proposed system where AU activation curves are dynamically modulated based on compound emotion semantics derived from GPT-4 analysis. This distinction forms the core experimental variable in subsequent evaluations.

- Classification Metrics for ResNet Emotion Classifier: Accuracy and F1-score were employed to evaluate the ResNet-based facial emotion recognition model trained on the AffectNet dataset. Three model variants (v1–v3) were compared to assess the effects of regularization, augmentation, and loss function strategies, as detailed in Section 4.1.
- AU Curve Metrics for Dynamic Synthesis Analysis: To assess differences between static (template-based) and dynamic (GPT-driven) facial expression generation, the following curve-level metrics were computed:
  – *Total Activation Energy*: The area under the AU activation curve, reflecting the cumulative intensity of expression over time.
  – *Spatial Distribution Pattern*: The number and diversity of activated AUs across emotional categories, indicating expression richness and anatomical dispersion.
  – *Temporal Evolution Structure*: Analysis of AU curve shapes (e.g., rise–hold–fall), peak timing, and duration to evaluate motion continuity and realism.
  These metrics are discussed in Section 4.2.
- Subjective Evaluation Metrics. Participants rated facial expressions along four perceptual dimensions—naturalness, emotional congruence, layering, and overall satisfaction—using a 5-point Likert scale. Each dimension was purposefully aligned with one of the research questions (RQs) defined in Section 1: Naturalness assessed the temporal smoothness and bio-fidelity of generated motion (RQ2); Emotional Congruenceevaluated the semantic alignment between facial expressions and spoken content (RQ3); Layering measured the perception of multiple coexisting emotional cues, serving as a perceptual proxy for compound emotion recognition (RQ1); and Satisfactionreflected the overall user preference. For statistical validation, we conducted a one-factor repeated-measures ANOVA (factor: System Type), followed by Bonferroni-corrected paired *t*-tests. Additional analysis included Pearson correlation and ANCOVA (covariates: age and 3D familiarity). Effect sizes were reported using partial $\eta^{2}$ and Cohen’s *d*.

## 4. Results

### 4.1. Results on Algorithm Architecture Output Analysis

Table 3 presents the ablation results on AffectNet. Variant $v_{1}$ attains a training accuracy of 90.4% but drops to 54.9% on the validation set, revealing severe over-fitting. Introducing dropout and a longer schedule in $v_{2}$ narrows the gap between training and validation and lifts the latter to 56.1% accuracy ($F_{1}=0.559$). The full regularization package in $v_{3}$—stronger augmentation, label smoothing, and AdamW with cosine-annealing—delivers the best generalization (62.6% validation accuracy, $F_{1}=0.625$) while keeping the training accuracy at a moderate 69.5%. Consequently, ResNet18 ( $v_{3}$) is adopted as the baseline emotion extractor for the downstream AU-based studies. This choice reflects a deliberate trade-off: while Transformer-based models may offer superior accuracy in multi-emotion tasks, our framework prioritizes low complexity to achieve real-time blending, as evidenced by the ablation results.

### 4.2. Results on AU Activation Pattern Analysis

To systematically evaluate the activation mechanisms of static and dynamic facial Action Units (AUs), this study conducted a comparative analysis of AU characteristics across three dimensions: (1) total activation energy, (2) spatial distribution pattern (activation heatmap), and (3) temporal dynamics. This analysis not only aids in understanding the differences in emotional expression between static and dynamic facial synthesis, but also provides key theoretical support for future emotion-driven animation systems.

#### 4.2.1. Total Activation Energy

**Figure 3:**
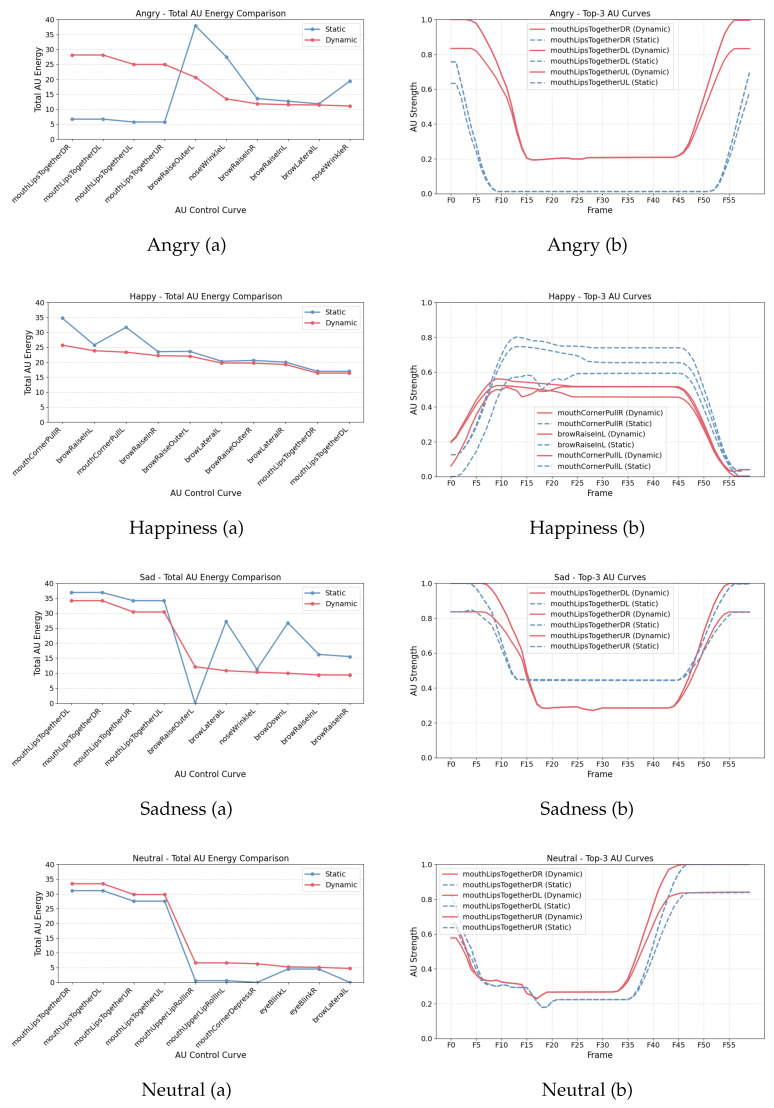
Comparison of AU activation and temporal progression across four emotions: (**a**) total AU activation energy across selected control curves; (**b**) dynamic evolution of the same AU curves over time (60 frames). Dynamic expressions show more sustained and distributed activation.

From an overall perspective, the distribution of AU activation energy under different emotional conditions revealed distinct advantages for both static and dynamic systems. As shown in Figure 3a, in the “happiness” condition, the static system exhibited stronger activation across several high-frequency AUs (e.g., mouthCornerPullR/L and browRaiseInL), with the top three AUs reaching an average total energy of 30.75, significantly higher than the dynamic system’s 24.31 (p<0.001, Cohen’s d>0.8). This suggests that static imagery may deliver a stronger perceptual impact in terms of peak expression amplitude.

However, the dynamic system showed significant advantages in several key mouth-related AUs, particularly under the “anger,” “neutral,” and “sadness” conditions. As presented in Table 4, the dynamic activation values for mouthLipsTogetherDL/DR/UL/UR in the “anger” condition were nearly four times higher than those of the static system, where DL, DR, UL, and UR denote the lower-left, lower-right, upper-left, and upper-right quadrants of the mouth region, respectively. with all Cohen’s *d* values exceeding 1.6 and p<0.001. In the “neutral” condition, AUs such as mouthCornerDepressR and mouthUpperLipRollInL/R also demonstrated significantly higher activation in the dynamic system, highlighting the synergistic enhancement of mouth-constricting actions within dynamic sequences.

These results indicate that although the static system can produce higher peak intensity in certain single-frame expressions, the dynamic system activates a broader range of mouth muscle groups over time and forms more realistic muscular coordination patterns. This leads to greater energy coverage and stronger expressive tension in facial animation.

#### 4.2.2. Spatial Distribution Pattern

**Figure 4:**
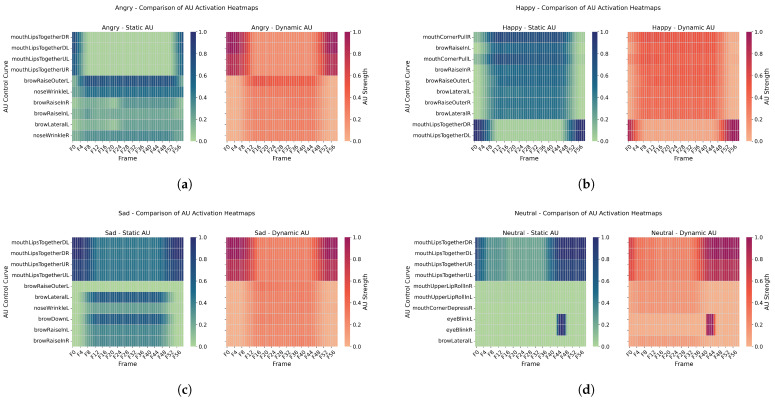
Spatial distribution patterns of AU activations under four emotional conditions. Each subfigure compares static (left) and dynamic (right) AU heatmaps for: (**a**) Anger, (**b**) Happiness, (**c**) Sadness, and (**d**) Neutral. Static activations are more concentrated in the upper facial region (e.g., brow and eyelid), while dynamic activations involve wider participation from the mouth, cheeks, and chin areas, especially in “Happiness” and “Neutral” expressions.

Analysis of AU heatmaps (Figure 4) revealed significant differences in spatial engagement between the two systems. The static system showed more concentrated AU activation in the upper facial region, primarily involving eyebrow elevation and eyelid compression. In contrast, the dynamic system exhibited more dispersed AU activation, with enhanced oral region engagement. This was especially evident under the “happiness” and “neutral” conditions, where a spatial topology of multi-region coordinated motion emerged. This distributional difference indicates that dynamic expressions more authentically replicate the multi-source-driven mechanisms of facial expression generation.

#### 4.2.3. Temporal Evolution Structure

In the temporal dimension, dynamic AU curves exhibited smoother and more continuous fluctuations, whereas static AU curves often displayed high-amplitude, single-peak activations, as shown in Figure 3b Taking the “sadness” emotion as an example, dynamic AUs followed a continuous pattern of “gradual rise–peak–gradual fall,” reflecting a more nuanced emotional delivery rhythm. In contrast, static AUs tended to produce abrupt peak activations within a limited number of frames, lacking transitional flow. This structural difference indicates that the dynamic system holds a significant advantage in simulating the gradualness and realism of emotional transitions.

In summary, the static AU model provides higher intensity and recognizability for capturing prototypical emotional moments, making it suitable for tasks requiring clearly labeled and goal-directed expression recognition. However, the dynamic AU activation structure demonstrates superior performance in the naturalness of emotional delivery, the completeness of spatial engagement, and the coherence of temporal sequencing. Particularly in scenarios involving compound emotion expression and micro-expression generation, dynamic curves enable more credible and fluid facial animations, offering critical support for emotion modeling in interactive systems such as digital humans.

### 4.3. Results on Subjective Evaluation Tests

#### 4.3.1. Main Effect of System Type

As shown in Table 5, the GPT-driven dynamic AU system obtained significantly higher scores than the static baseline on all four subjective dimensions—naturalness, emotional congruence, expression layering, and satisfaction (all p<0.001; partial $\eta^{2}=0.344–0.507$). The largest gain appeared in Naturalness [F(1,50)=42.21, partial $\eta^{2}=0.507$], followed by Layering. The latter serves as a perceptual proxy for compound-emotion recognition: higher layering scores indicate that observers perceived multiple co-occurring affects in the dynamic expressions.

Only Naturalness exhibited a significant SystemType × Age interaction [F(5,41)=3.62, p=0.008, partial $\eta^{2}=0.306$]; the other dimensions showed no interaction effects.

**Figure 5:**
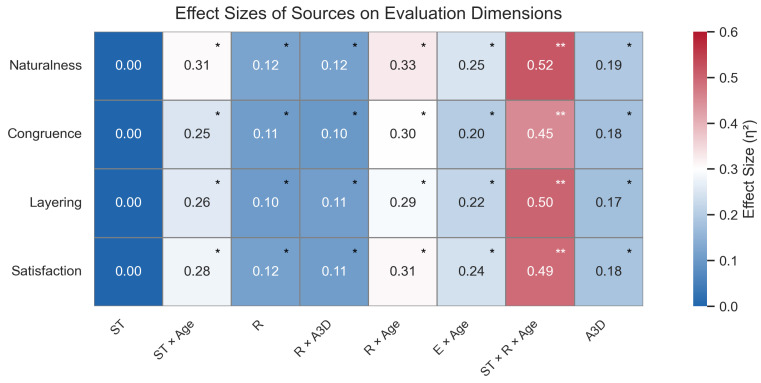
Partial $\eta^{2}$ effect size heatmap of different predictors and interactions on four subjective ratings. * *p* < 0.05, ** *p* < 0.01. Note. ST = SystemType; A3D = Acceptance of 3D Characters; R = Ratings (Naturalness, Congruence, Layering, Satisfaction); Age = Participant Age Group.

To comprehensively present the explanatory power of different independent variables and their interactions across the four subjective dimensions, a heatmap of partial $\eta^{2}$ effect sizes was generated. As shown in Figure 5, system type exhibited medium to large effect sizes across all dimensions, while other variables (e.g., Emotion, Ratings, Age) and certain interaction terms showed substantial variation in effect sizes across different dimensions. This further underscores the role of system type as the primary determining factor.

#### 4.3.2. Paired-Sample Comparison

Further results from the paired-sample *t*-tests (Table 6) revealed significant differences in the mean scores across all four rating dimensions between the two system types (all *p* < 0.001), with all differences in the same direction—scores for the dynamic system were significantly higher than those for the static system. Expression layering (*t* = −10.62, d = −1.49) and satisfaction (*t* = −9.43, d = −1.32) exhibited very large effect sizes, indicating that participants showed a clear preference for the richness and overall perception of compound expressions generated by the dynamic system.

#### 4.3.3. Effects of Age and Technology Acceptance

**Figure 6:**
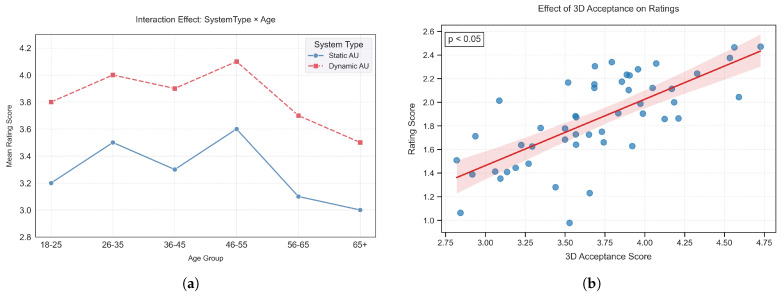
Moderating effects on naturalness ratings: (**a**) Interaction plot of Age × System Type on Naturalness ratings. (**b**) Covariate effect of 3D Acceptance on ratings, where blue dots represent individual participant scores, the red line indicates the fitted regression line, and the shaded area shows the 95% confidence interval.

In the dimension of naturalness, further analysis revealed a significant interaction between system type and age (*p* = 0.008), with a moderate interaction effect size (partial $\eta^{2}$ = 0.306). As shown in Figure 6a, participants across all age groups showed a preference for the dynamic system, with the 26–55 age group exhibiting the most pronounced rating difference and the 65+ group showing the smallest difference.

In addition, technology acceptance demonstrated a significant main effect on naturalness ratings (F = 9.83, *p* = 0.003, $\eta^{2}$ = 0.19). As illustrated in Figure 6b, the higher the user’s overall acceptance of the 3D system, the higher their rating of expression naturalness.

#### 4.3.4. Inter-Dimensional Correlation Analysis

**Figure 7:**
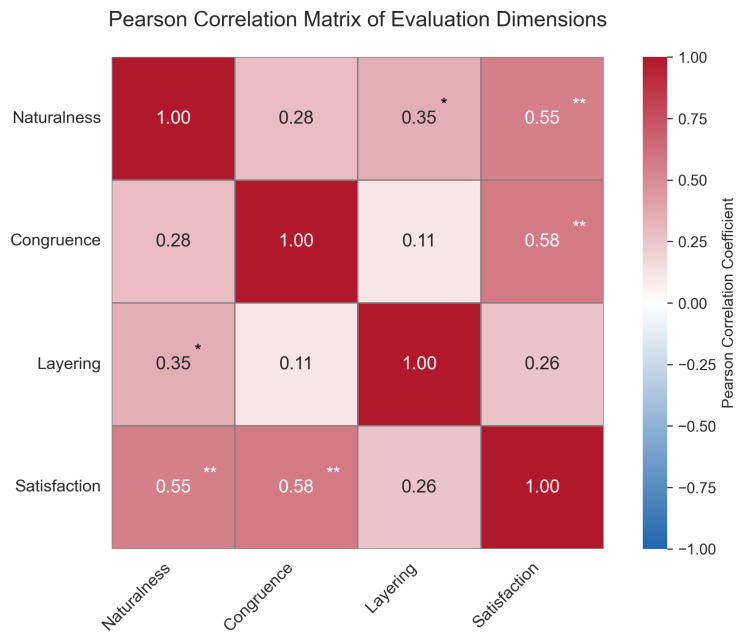
Heatmap showing the Pearson correlation coefficients between four subjective rating dimensions: Naturalness, Congruence, Layering, and Satisfaction. Asterisks indicate significance levels (* p<0.05, ** p<0.01).

Pearson correlation analysis revealed stable structural relationships among the four subjective dimensions, as shown in Figure 7. The highest correlation was observed between emotional congruence and satisfaction (r = 0.578, *p* < 0.01), followed by naturalness and satisfaction (r = 0.552, *p* < 0.01), both indicating significant positive correlations of moderate or greater strength. These results suggest that semantic congruence is a primary determinant of user satisfaction, while naturalness plays a key role in visual acceptability and perceived realism.

**Figure 8:**
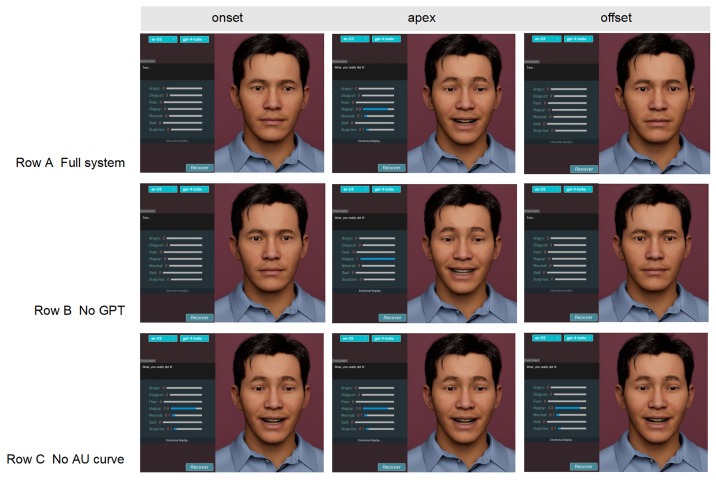
Qualitative impact of key modules. Row A: full system; Row B: no GPT (uniform AU weights); Row C: no AU-curve driver. Columns show onset, apex and offset frames of the utterance “I’m so excited to see you!”. A miniature bar chart beside each frame visualizes the seven-class emotion intensity, highlighting the loss of surprise components without GPT (Row B) and the temporal jerk introduced when curve modulation is removed (Row C).

As illustrated in Figure 8, removing GPT suppresses surprise-related AUs, while discarding the AU-curve driver introduces abrupt on/off transitions. The full configuration therefore achieves both context-appropriate muscle engagement and smooth temporal dynamics.

### 4.4. Statistical Analysis

#### Correlation Analysis Between Subjective Ratings and Objective AU Features

To be specific, the study constructed correlation matrices based on Ekman’s basic emotions under both static and dynamic AU systems. AU activation features were quantified using the average value of control curves over the video duration, while subjective ratings were derived from participants’ scores on a four-dimensional Likert scale. Pearson correlation coefficients were computed and visualized through heatmaps to reveal the coupling relationships between objective AU activations and subjective perceptual dimensions.

**Figure 9:**
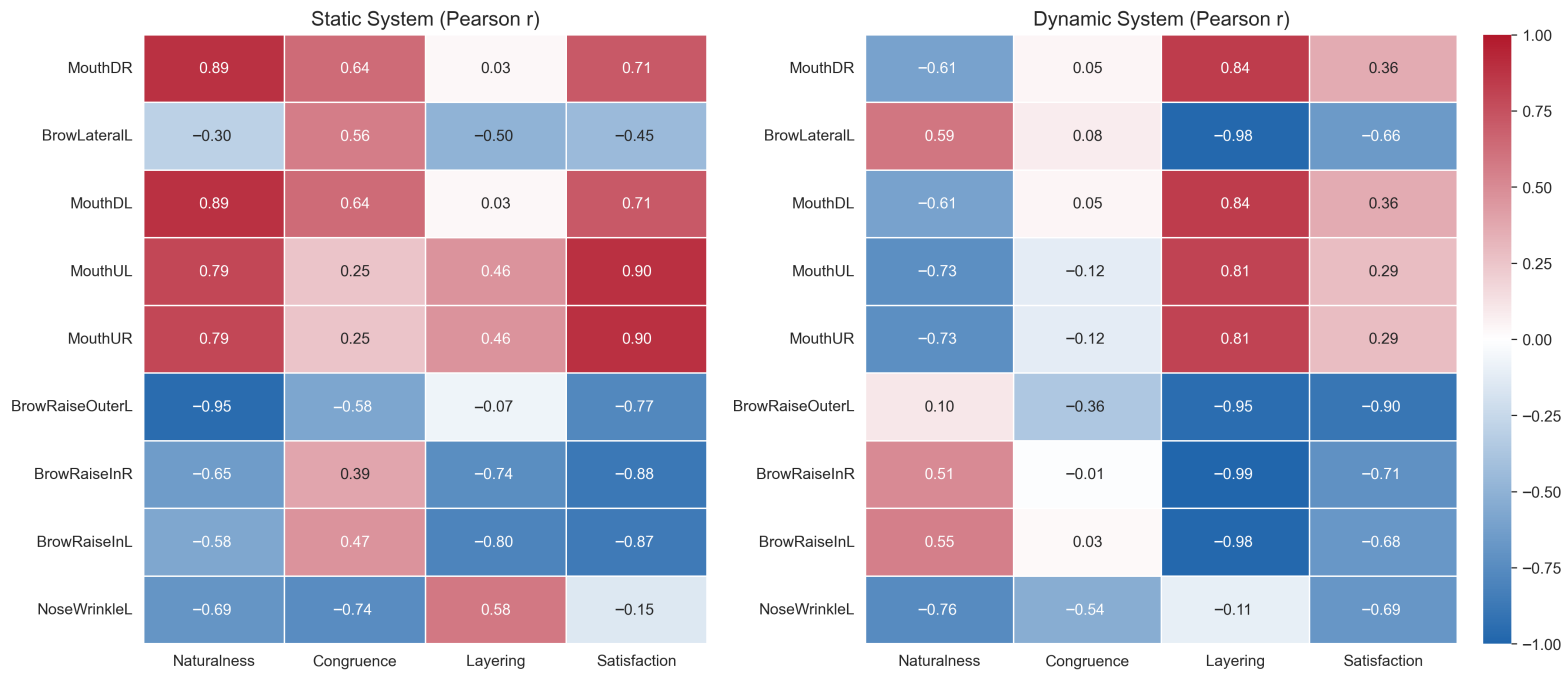
Pearson correlation heatmaps between AU activation features and subjective perceptual ratings across static (**left**) and dynamic (**right**) systems. The color indicates the strength and direction of correlation (from −1.0 to +1.0) between each AU curve and four subjective dimensions: naturalness, emotional congruence, layering, and satisfaction.

As shown in Figure 9, exaggerated activations of specific AU curves—such as mouthLipsTogether and noseWrinkle—in the static system were strongly negatively correlated with perceived naturalness and emotional congruence. For instance, mouthLipsTogetherUR showed a correlation coefficient of −0.90 with congruence, while noseWrinkleR reached −0.99 (*p* = 0.009), indicating that excessive muscle contractions may diminish perceived naturalness and emotional congruence. In contrast, browRaiseOuterL demonstrated significant positive correlations with layering (r = 0.91) and satisfaction (r = 0.64), suggesting that subtle motion signals may enhance expression richness and user acceptance.

In the dynamic system, similar trends were more pronounced. The aforementioned exaggerated AUs remained negative indicators, with correlations ranging from moderate to strong with naturalness and satisfaction (e.g., noseWrinkleR and naturalness, r = −0.94). Meanwhile, movements such as browRaiseIn exhibited strong positive correlations with satisfaction and layering, highlighting their beneficial role in conveying smooth emotional transitions.

Although high-confidence significance testing was limited by the small sample size (n = 8), these preliminary findings suggest potential mechanisms by which different AU activation patterns influence subjective user experience: natural, subtle, and gradual AU activations are more likely to yield positive perceptual evaluations, whereas large-amplitude, abrupt activations may be perceived as unnatural or incongruent.

### 4.5. Real-Time Performance Evaluation

A representative end-to-end run on an Intel^®^ Core^TM^ i7-14700KF/RTX 4070 SUPER workstation yields a total dialog latency of 2.47 s: 0.62 s for local speech-to-text conversion, 1.84 s for GPT-4 prompt construction and AU parsing, and only 0.01 s for BlendShape update with Unreal Engine 5.3 (UE5.3) rendering (Table 7). Peng et al. [38] report that users tolerate delays up to 4 s in conversational interactions, whereas satisfaction deteriorates sharply beyond 8 s. Our latency therefore falls well within the empirically accepted window, and the renderer maintains ∼107 FPS (9.32 ms per frame), guaranteeing visually smooth feedback. Consequently, the proposed framework fulfills both conversational and visual real-time requirements.

## 5. Discussion

This section revisits the four research questions (RQs) posed in the Introduction, relates our findings to prior studies, and analyzes the contribution of each module in the proposed pipeline.

Compound-emotion inference. Subjective results in Table 5 confirm that the GPT-driven dynamic system conveys blended affect more convincingly than the static template. Naturalness increases from 3.31 ± 0.94 to 4.02 ± 0.80, and layering from 3.33 ± 0.91 to 4.09 ± 0.72, with large effect sizes (p<0.001). These gains indicate that language-conditioned AU re-weighting delivers perceptually richer expressions than a fixed AU set.

Static versus dynamic synthesis. As illustrated in Figure 3, static AU templates yield abrupt, single-peak profiles, whereas the dynamic strategy produces smoother rise–peak–fall curves and engages a wider set of action units—especially in “happiness” and “neutral” clips. The observation supports the hypothesis that semantic re-weighting enhances motion realism without introducing additional temporal latency.

Semantic-to-AU mapping. Analysis in Section 4.2 shows that dynamic AU trajectories exhibit higher dispersion across facial regions than their static counterparts, demonstrating that GPT-derived semantic weights translate into behaviorally meaningful AU activation patterns.

Overall performance. Within the visual branch, ResNet18-V3 achieves the highest accuracy (0.626) and balanced $F_{1}$ (0.625) among the backbones we tested, while retaining an offline inference time of 1.31 ms (Table 1). Subjectively, the dynamic mode outperforms the static baseline across naturalness, emotional congruence, layering and satisfaction, with partial $\eta^{2}$ ranging from 0.344 to 0.507.

Module influence and alternatives. ResNet18-V3 offers the best accuracy-latency trade-off among our benchmarks; MobileNetV2 reduces model size but loses 12% $F_{1}$, whereas ResNet50 doubles parameters without gain. The BlendShape + state-machine driver integrates seamlessly with Unreal Engine and requires no per-subject calibration, unlike physics-based or NeRF rigs. GPT-4 provides context sensitivity that VAD lexicons and 13 B open-source LLMs cannot match. The selected trio therefore achieves the most pragmatic balance between accuracy, expressiveness, and engineering cost.

## 6. Conclusions

### 6.1. Key Findings and Contributions

This study presents a closed-loop emotion expression system that spans from semantic parsing to 3D facial expression synthesis, bridging the response pathway between language understanding and facial muscle activation. The system introduces a multimodal emotion recognition framework integrating text, speech, and facial images and builds a robust emotion classifier based on a ResNet18 backbone. In the expression synthesis module, the system utilizes Unreal Engine to finely control AU motion trajectories, successfully generating expression animation sequences with structural continuity and high naturalness.

Furthermore, the dynamic AU weight allocation mechanism driven by GPT-based semantic outputs enables the generation of compound emotional expressions, surpassing the limitations of conventional rule-based systems that rely on one-to-one mappings for single emotions. User experiments further demonstrated that the dynamic expressions generated by the system received high ratings across multiple perceptual dimensions and exhibited significant correlations with AU activation parameters, confirming a structural alignment between affect generation and human perception.

### 6.2. Limitations and Future Work

Despite its strong performance across various metrics, the current system still faces several limitations. First, the emotion categories are restricted to Ekman’s seven basic emotions, without incorporating more complex, socially- or culturally-grounded, or blended emotion expressions [39,40], which limits adaptability in realistic interactive contexts. Second, although the semantic outputs of the GPT model have been standardized, their stability under ambiguous or colloquial language input remains to be further validated [41].

Future work will consider incorporating large-scale pretrained multimodal models to improve generalization to complex input scenarios. Additionally, methods such as neural deformation networks may be employed to enhance personalization and fine-grained control in facial expression synthesis. Expanding the participant sample size and including culturally diverse groups will also be essential to explore how individual differences affect system acceptance and perception of expressions.

## Figures and Tables

**Table 1:** Performance Comparison of Classification Models.

Model	$F_{1}$-Score	Accuracy	Inference Time (ms)	Model Size (MB)
MLP [19]	0.028	0.125	2.16	24.02
MobileNetV2 [17,18]	0.505	0.504	5.85	**8.52**
**ResNet18 V3-Opt** [20,21]	**0.625**	**0.626**	**1.31**	42.65
ResNet50 [22,23]	0.539	0.536	7.76	89.70

Note: Boldface highlights the best performance.

**Table 2:** ResNet18-Based Emotion Classifier Architecture.

Layer	Kernel Configuration	Output Dimension
Input	RGB image: 3×224×224	3×224×224
Conv1	7×7, 64 filters, stride =2	64×112×112
MaxPooling (optional)	3×3, stride =2	64×56×56
ResBlock1	3×3, 64 × 2	64×56×56
ResBlock2	3×3, 128 × 2	128×28×28
ResBlock3	3×3, 256 × 2	256×14×14
ResBlock4	3×3, 512 × 2 (fine-tuned)	512×7×7
Global Avg. Pooling	AdaptiveAvgPool2d →1×1	512×1
Fully Connected (FC)	Linear (512 → 7)	7 logits
Softmax Activation	Softmax over 7 logits	7-dimensional probability
Output	argmax(Softmax)→ predicted label	Label + confidence vector

**Table 3:** Ablation Study of ResNet18 Variants on AffectNet.

Variant	Data Augmentation	Loss Function	Optimizer + Scheduler	Train Acc (%)	Val Acc (%)	Val F1
ResNet18 $v_{1}$	Flip, rotate, jitter	CrossEntropy	Adam	**90.44**	54.90	0.5438
ResNet18 $v_{2}$	Same as v1	CrossEntropy	Adam	59.72	56.14	0.5590
ResNet18 $v_{3}$	Flip, rotate, affine, color	CE + Label Smoothing	AdamW + CosineAnnealing	69.49	**62.57**	**0.6254**

*v*_1_ exhibits overfitting. *v*_2_ adds dropout. *v*_3_ achieves best generalization via strong augmentation and regularization. **Bold** values indicate the best performance in each column.

**Table 4:** Comparison of Top 5 AU Activation Energies between Dynamic and Static Expressions across Emotions.

Emotion	AU Name	Dyn. Energy	Stat. Energy	*p*-Value	Cohen’s *d*
Angry	mouthLipsTogetherDL	28.15	6.69	<0.001	1.64
	mouthLipsTogetherDR	28.15	6.69	<0.001	1.64
	mouthLipsTogetherUL	24.99	5.73	<0.001	1.84
	mouthLipsTogetherUR	24.99	5.73	<0.001	1.84
	browRaiseOuterL	20.67	37.94	<0.001	−2.11
Happiness	mouthCornerPullR	25.71	34.76	<0.001	−1.18
	mouthCornerPullL	23.37	31.70	<0.001	−1.18
	browRaiseInL	23.85	25.79	0.005	−0.37
	browRaiseInR	22.20	23.55	0.029	−0.29
	browRaiseOuterL	22.04	23.63	0.010	−0.34
Sadness	browRaiseOuterL	12.10	0.00	<0.001	1.43
	browLateralL	10.78	27.18	<0.001	−1.96
	browDownL	9.92	26.80	<0.001	−1.96
	browRaiseInL	9.35	16.22	<0.001	−1.89
	mouthLipsTogetherDL	34.19	36.93	0.007	−0.36
Neutral	mouthUpperLipRollInL	6.61	0.56	<0.001	1.39
	mouthUpperLipRollInR	6.61	0.57	<0.001	1.39
	mouthCornerDepressR	6.30	0.00	<0.001	1.41
	mouthLipsTogetherDL	33.45	31.10	<0.001	0.48
	mouthLipsTogetherDR	33.45	31.10	<0.001	0.48

Note. DL = down left, DR = down right, UL = upper left, UR = upper right; L = left, R = right (relative to the viewer’s perspective).

**Table 5:** Main Effects of System Type and Its Interaction with Age on Four Subjective Ratings.

Ratings	Mean (Static)	Mean (Dynamic)	F	*p*-Value	Partial $\eta^{2}$	Significant Interaction
Naturalness	3.31 ± 0.94	4.02 ± 0.80	42.21	<0.001	0.507	SystemType × Age (*p* = 0.008)
Congruence	3.41 ± 0.89	4.12 ± 0.73	34.78	<0.001	0.459	–
Layering	3.33 ± 0.91	4.09 ± 0.72	39.65	<0.001	0.492	–
Satisfaction	3.31 ± 0.93	3.95 ± 0.82	21.45	<0.001	0.344	–

Note. Values are presented as mean ± standard deviation. Repeated measures ANOVA was used. Only Naturalness showed a significant SystemType × Age interaction, *F*(5,41) = 3.619, *p* = 0.008, partial $\eta^{2}$ = 0.306.

**Table 6:** Paired Samples *t*-Test Results: Static vs. Dynamic System Ratings Across Four Dimensions.

Dimension	Static (M ± SD)	Dynamic (M ± SD)	*t*	df	*p* (2-Tailed)	Cohen’s *d*	95% CI (*d*)
Naturalness	3.54 ± 0.54	3.94 ± 0.41	−4.43	50	<0.001	−0.62	[−0.92, −0.32]
Congruence	3.44 ± 0.61	3.80 ± 0.61	−4.70	50	<0.001	−0.66	[−0.96, −0.35]
Layering	3.20 ± 0.42	4.08 ± 0.43	−10.62	50	<0.001	−1.49	[−1.88, −1.08]
Satisfaction	3.45 ± 0.41	4.11 ± 0.36	−9.43	50	<0.001	−1.32	[−1.69, −0.94]

Note. Paired samples *t*-tests were conducted between static and dynamic systems. Negative *t* and Cohen’s *d* values indicate higher ratings for the dynamic system. Effect sizes ranged from medium to very large.

**Table 7:** End-to-end latency of the real-time emotional-response pipeline.

Step	Processing Module	Mean Latency (s)	Description
1	Audio capture and speech-to-text (TTS)	0.62	Microphone input to text transcription
2	GPT-4 prompt and AU parsing	1.84	Prompt construction, LLM inference, AU weight parsing
3	BlendShape update and UE5 rendering	0.01	State-machine drive, shape blending, first frame render
Total dialog latency		2.47	Speech input to first facial frame
Supplement: frame-render speed		9.32 ms/frame (approx. 107 FPS)	Sustained UE5 performance

Test platform: Intel(R) Core(TM) i7-14700KF CPU, NVIDIA GeForce RTX 4070 SUPER GPU, 64 GB RAM, Windows 11.

## Data Availability

Supplementary Materials (Table S1) contain the mapping of facial Action Units (AUs) to control curves used in the animation system. Further inquiries can be directed to the corresponding author.

## Supplementary Materials

The following supporting information can be downloaded at: https://www.mdpi.com/article/10.3390/s25165117/s1, Table S1: Mapping of Action Units (AUs) to 3D control curve names for facial animation.
