# Supplementary material for: Development of an Interactive Digital Human with Context-Sensitive Facial Expressions
Source: Sensors (Basel). 2025 Aug 18;25(16):5117. doi: 10.3390/s25165117 (PMC12390055; doi:10.3390/s25165117)
Supplement: Supplementary file 1 [file sensors-25-05117-s001.zip › sensors-3724219-supplementary.pdf]

Mapping of Action Units (AUs) to 3D control curve names for facial animation.

Action Unit	Description	Control Curves	Image
AU1	inner brow raiser	CTRL_L_brow_raiseIn<br>CTRL_R_brow_raiseIn	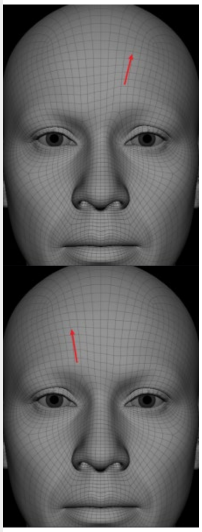
AU2	outer brow raiser	CTRL_L_brow_raiseOut<br>CTRL_R_brow_raiseOut	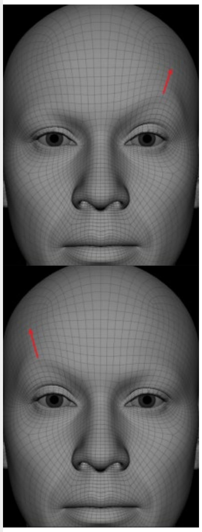
AU4	brow lowerer	CTRL_L_brow_down<br>CTRL_R_brow_down<br>CTRL_L_brow_lateral<br>CTRL_R_brow_lateral	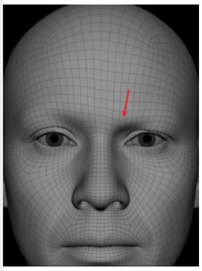

			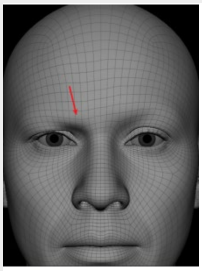 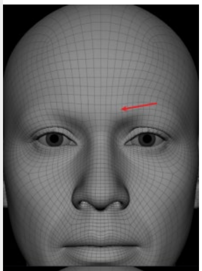 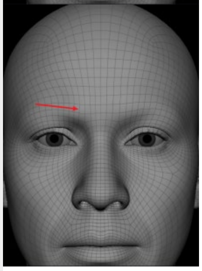
AU5	upper lid raiser	CTRL_L_eye_eyelidU<br>CTRL_R_eye_eyelidU	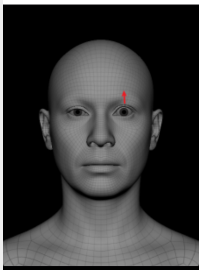 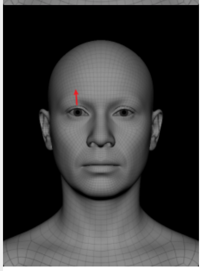
AU6	cheek raiser	CTRL_L_eye_cheekRaise<br>CTRL_R_eye_cheekRaise	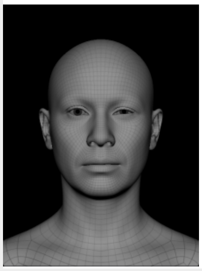

			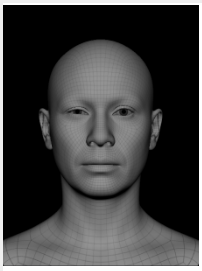
AU7	lid tightener	CTRL_L_eye_squintInner<br>CTRL_R_eye_squintInner	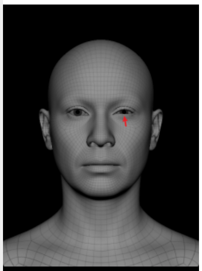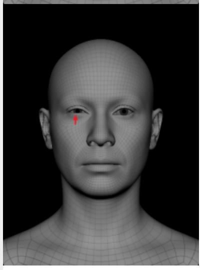
AU9	nose wrinkler	CTRL_L_nose<br>CTRL_R_nose<br>CTRL_R_nose_wrinkleUpper<br>CTRL_L_nose_wrinkleUpper	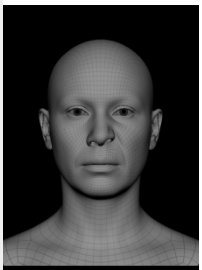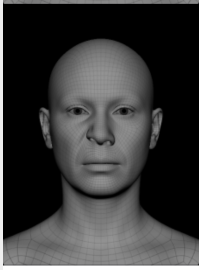
AU10	upper lip raiser	CTRL_L_mouth_upperLipRaise<br>CTRL_R_mouth_upperLipRaise	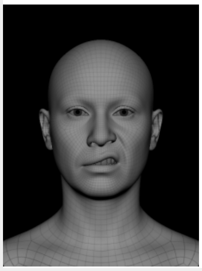

			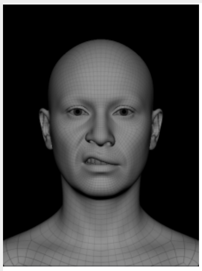
AU11	nasolabial furrow deepener	CTRL_L_nose_nasolabialDeepen<br><br>CTRL_R_nose_nasolabialDeepen	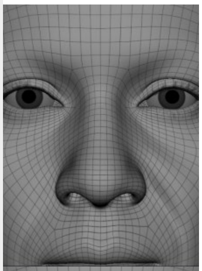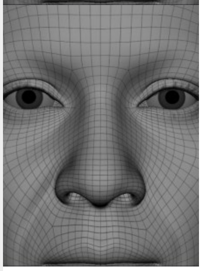
AU12	lip corner puller	CTRL_L_mouth_cornerPull<br><br>CTRL_R_mouth_cornerPull	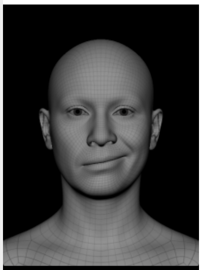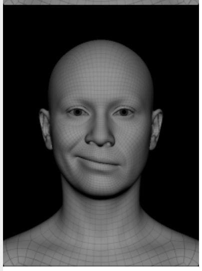
AU13	sharp lip puller	CTRL_L_mouth_sharpCornerPull<br><br>CTRL_R_mouth_sharpCornerPull	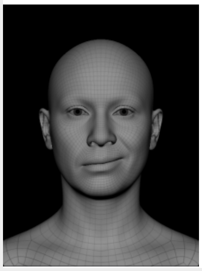

			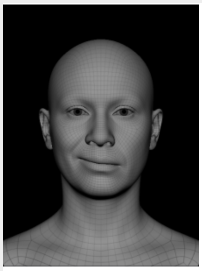
AU14	dimpler	CTRL_L_mouth_dimple<br>CTRL_R_mouth_dimple	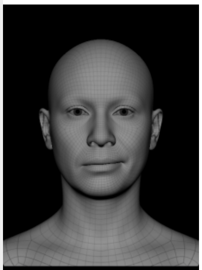<br>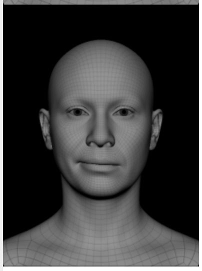
AU15	lip corner depressor	CTRL_L_mouth_cornerDepress<br>CTRL_R_mouth_cornerDepress	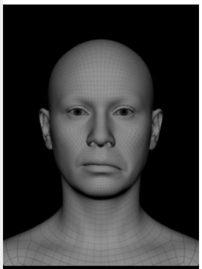<br>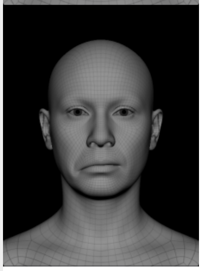
AU16	lower lip depressor	CTRL_L_mouth_lowerLipDepress<br>CTRL_R_mouth_lowerLipDepress	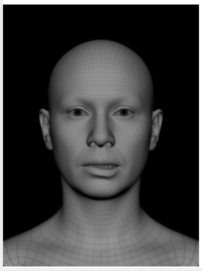

			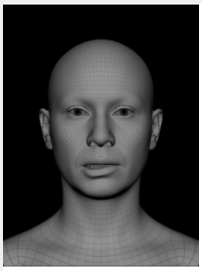
AU17	chin raiser	CTRL_L_jaw_ChinRaiseD<br>CTRL_R_jaw_ChinRaiseD	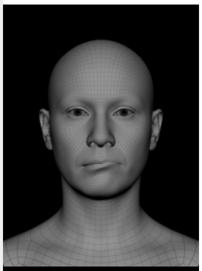<br>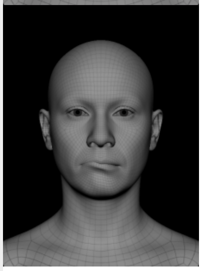
AU18	lip pucker	CTRL_L_mouth_purseU<br>CTRL_R_mouth_purseU<br>CTRL_L_mouth_purseD<br>CTRL_R_mouth_purseD<br>CTRL_L_mouth_lipsTowardsTeethU<br>CTRL_R_mouth_lipsTowardsTeethU<br>CTRL_L_mouth_lipsTowardsTeethD<br>CTRL_R_mouth_lipsTowardsTeethD	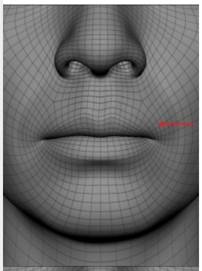<br>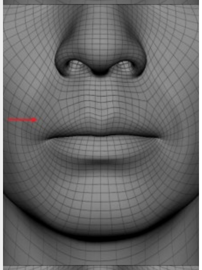<br>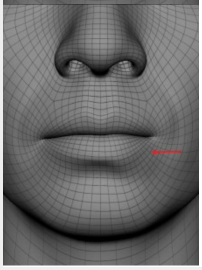

			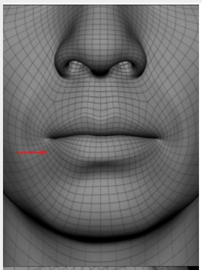<br>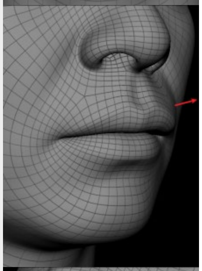<br>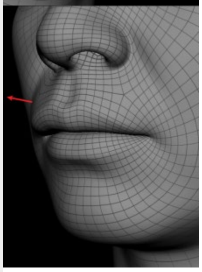<br>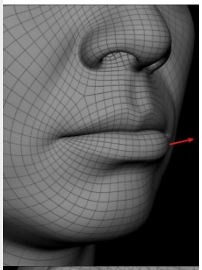<br>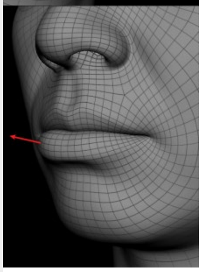
AU20	lip stretcher	CTRL_L_mouth_stretch<br>CTRL_R_mouth_stretch	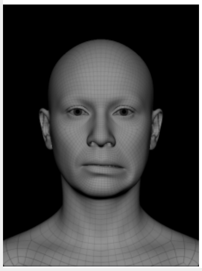

			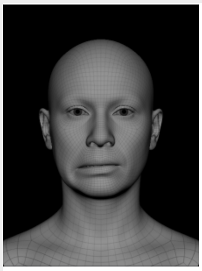
AU22	lip funneler	CTRL_L_mouth_funnelU<br>CTRL_R_mouth_funnelU<br>CTRL_L_mouth_funnelD<br>CTRL_R_mouth_funnelD	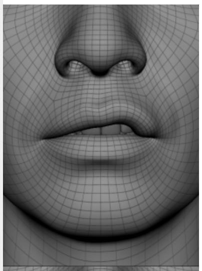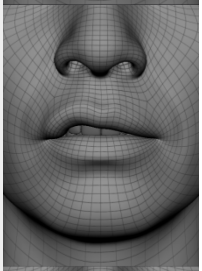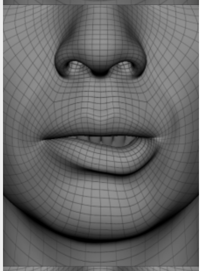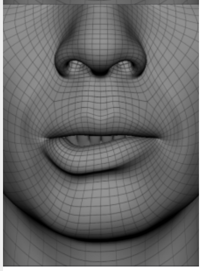

AU23	lip tightener	CTRL_L_mouth_tightenU<br>CTRL_R_mouth_tightenU<br>CTRL_L_mouth_tightenD<br>CTRL_R_mouth_tightenD	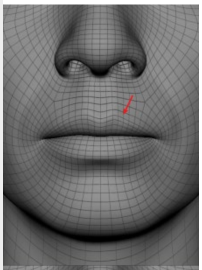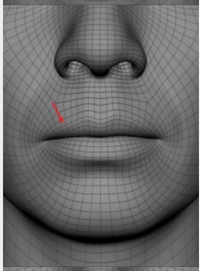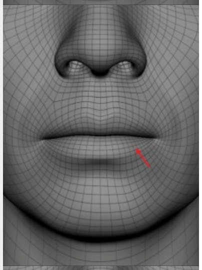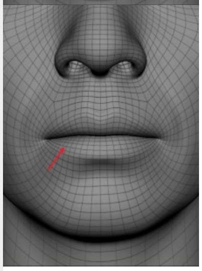
AU27	mouth stretch	CTRL_C_jaw	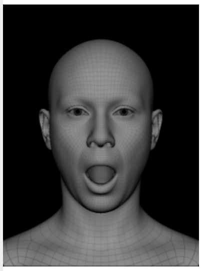
AU28	lip presser	CTRL_L_mouth_lipsPressU<br>CTRL_R_mouth_lipsPressU<br>CTRL_L_mouth_lipsPressD<br>CTRL_R_mouth_lipsPressD	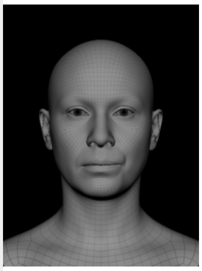

			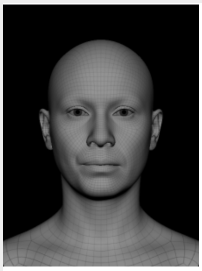
AU29	jaw thurst	CTRL_C_jaw_fwdBack	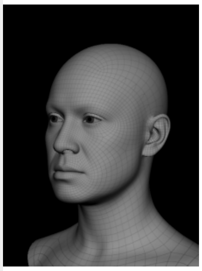
AU34	puff	CTRL_L_mouth_suckBlow<br>CTRL_R_mouth_suckBlow	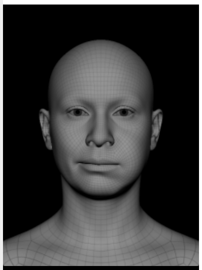<br>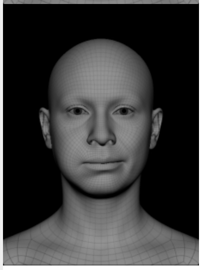
AU43	eye closure	CTRL_R_eye_blink<br>CTRL_L_eye_blink	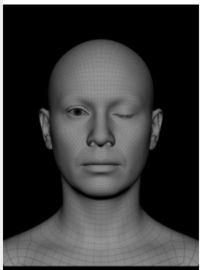<br>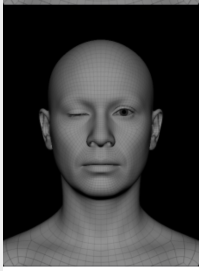
AU45	blink		

AU63	eyes up	CTRL_L_eye<br><br>CTRL_R_eye	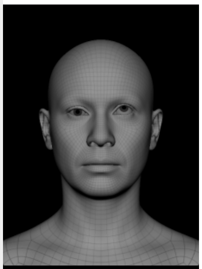<br>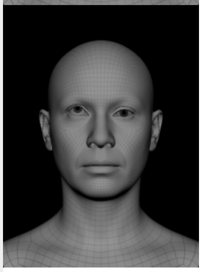

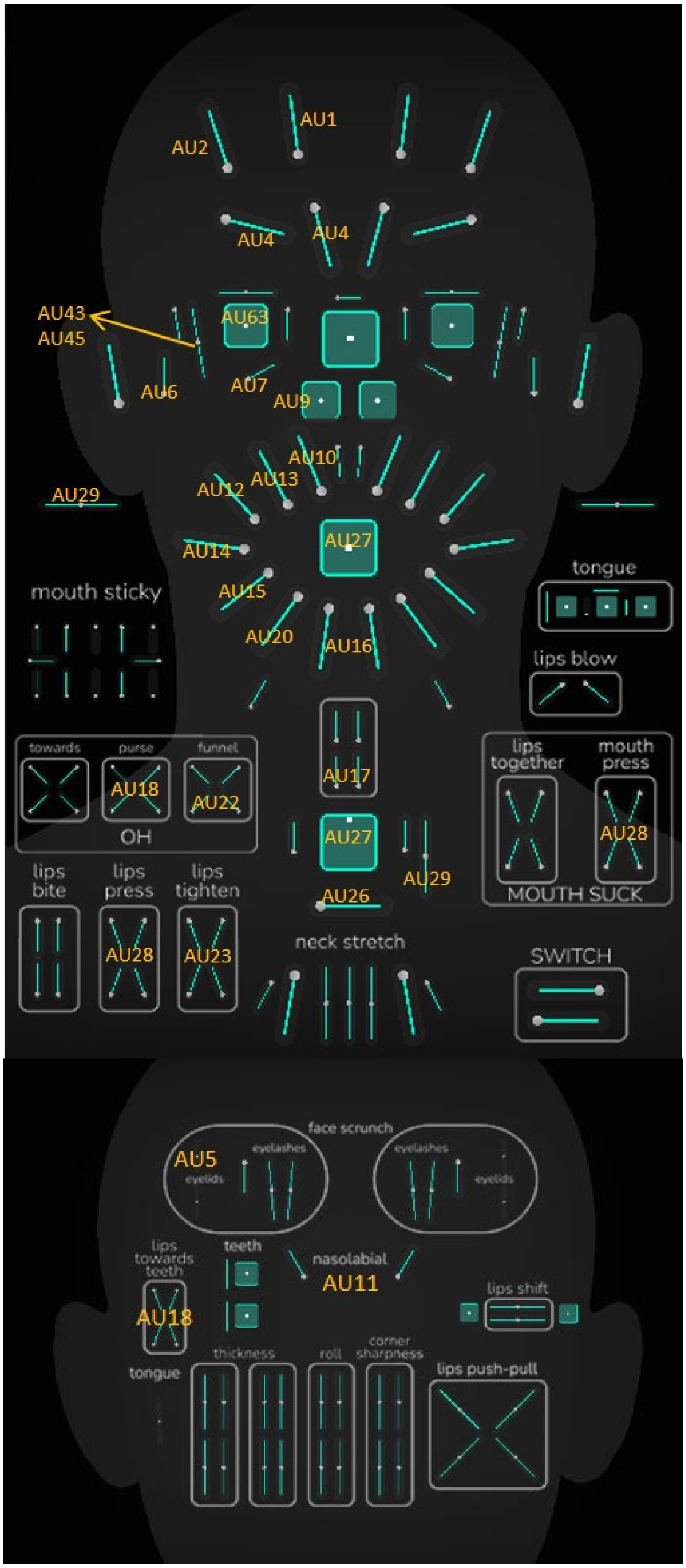
AU1
AU2
AU4
AU4
AU43
AU45
AU63
AU6
AU7
AU9
AU10
AU12
AU13
AU29
AU27
AU14
mouth sticky
AU15
tongue
AU20
AU16
lips blow
towards
purse
funnel
AU18
AU22
OH
AU17
lips together
mouth press
AU28
AU27
AU29
MOUTH SUCK
lips bite
lips press
lips tighten
AU26
AU28
AU23
neck stretch
SWITCH
face scrunch
AU5
eyelashes
eyelids
eyelashes
eyelids
lips towards teeth
teeth
nasolabial
AU11
lips shift
AU18
thickness
roll
corner sharpness
tongue
lips push-pull
